# A Multidrug and Toxic Compound Extrusion Transporter, RgMATE6, Facilitates Vacuolar Transport of Acteoside in *Rehmannia glutinosa*

**DOI:** 10.3390/plants14233608

**Published:** 2025-11-26

**Authors:** Yanhui Yang, Yuying Li, Yuxuan Wang, Mingjie Li, Zhongyi Zhang, Ruifang Li, Weiwei Wang, Fuxi Shen, Mengman Yan

**Affiliations:** 1 School of Bioengineering, Henan University of Technology, Lianhua Street 100, Zhengzhou High-Technology Zone, Zhengzhou 450001, China; 2022920187@stu.haut.edu.cn (Y.L.); 241060100324@stu.haut.edu.cn (Y.W.); lrf@haut.edu.cn (R.L.); 231100100424@stu.haut.edu.cn (W.W.); 231170400220@stu.haut.edu.cn (F.S.); 221060300128@stu.haut.edu.cn (M.Y.); 2 College of Bee Science and Biomedicine, Fujian Agriculture and Forestry University, Jinshan Road, Cangshan District, Fuzhou 350002, China; limingjie@fafu.edu.cn (M.L.); hauzzy@163.com (Z.Z.)

**Keywords:** acteoside, MATE, production, *Rehmannia glutinosa*, transport activity, vacuole

## Abstract

Acteoside (ACT), a prominent compound of the hydroxytyrosol-type phenylethanol glycoside (HPG) class, is present in plants and holds significant potential for food and pharmaceutical applications. However, the limited production of ACT in plants restricts its broader utilization. Although the biosynthetic pathways of ACT are increasingly understood, its transport mechanisms within plants remain unclear. RgMATE6, a vacuolar-type Multidrug and Toxic Compound Extrusion (MATE) transporter identified in *Rehmannia glutinosa* (a plant known for ACT p roduction), was selected for investigation. This study aims to elucidate the role of RgMATE6 in ACT transport and its impact on ACT biosynthesis. Our study utilized a multidisciplinary approach, including in silico analysis to predict substrate specificity, quantitative real-time PCR (qRT-PCR) to quantify gene expression, HPLC to measure HPG levels, vacuolar membrane vesicle uptake assays to validate RgMATE6 transport activity in vitro, and genetic transformation in *R. glutinosa* to assess its functional roles in vivo. In silico analysis identified RgMATE6 as a phenolic compound transporter, and correlation analysis revealed a strong positive association between the HPG accumulation and *RgMATE6* expression in *R. glutinosa*. Functional validation through vacuolar membrane vesicle uptake assays in *Nicotiana benthamiana* confirmed RgMATE6’s role as an HPG transporter, demonstrating a significant preference for ACT. Overexpression and repression experiments in *R. glutinosa* further demonstrated that RgMATE6 facilitates ACT import into vacuoles and enhances its production. Additionally, tissue-specific expression analysis revealed the coordinated expression patterns between *RgMATE6* and six ACT biosynthetic genes in the transgenic plants. RgMATE6 facilitates the transport and accumulation of ACT within vacuoles, and its expression might synergize with ACT biosynthesis. These findings establish a framework for improving ACT and other HPG production through targeted manipulation of plant MATE transporters.

## 1. Introduction

Hydroxytyrosol-type phenylethanol glycosides (HPGs) constitute a significant group of naturally occurring phenolic compounds. Encompassing hundreds of distinct molecules, HPGs are found in a wide range of plants and herbs [1,2]. Research has documented their diverse biological effects, which include antioxidant, antimicrobial, and neuroprotective activities [3,4,5]. Structurally, HPGs are characterized by a hydroxyphenylethyl core linked to a β-glucopyranose unit, often decorated with aromatic acids or various sugars via ester or glycosidic bonds [6,7]. Among HPGs, acteoside (ACT) stands out as a well-known bioactive compound with a hydroxysalidroside moiety conjugated to caffeoyl and rhamnose groups. This compound is a major pharmacological constituent in several medicinal plants, including *Rehmannia glutinosa* [8,9], *Cistanche tubulosa* [10], and *Sesamum indicum* [11]. Renowned for its multifunctional health benefits, ACT exhibits broad-spectrum therapeutic potential against inflammatory, neoplastic, and infectious pathologies [12,13,14,15]. However, the limited production of ACT in plants presents a major bottleneck for its industrial applications, necessitating the development of effective strategies to enhance its biosynthesis and accumulation [9,16].

The content of ACT in plants, like other plant metabolites, depends on its biosynthesis, transport, and accumulation processes [9,16]. Its biosynthesis route starts from phenylalanine and tyrosine, precursors generated by the cytosolic shikimate pathway [8,9,17]. While significant progress has been made in elucidating the shared upstream biosynthetic pathways of HPGs [18,19,20,21], recent research has also revealed the downstream, specific branch metabolic pathways involved in ACT biosynthesis in plants [7,16,22]. However, the mechanisms governing the transport and accumulation of ACT within plant cells remain largely unknown.

Plant vacuoles function as dynamic reservoirs for secondary metabolites, the accumulation and distribution of which are tightly controlled by transmembrane transporters [23,24,25]. Among these, the Multidrug and Toxic Compound Extrusion (MATE) family of transporters has emerged as a major player in the vacuolar import of specialized metabolites, including phenolic compounds and glycosides [25,26,27]. MATE proteins, typically 400–550 amino acids in length and containing 9–12 transmembrane helices, mediate H^+^/Na^+^-coupled antiport activity to facilitate metabolite sequestration into vacuoles [28,29,30]. Functional characterization of MATE transporters in species such as *Arabidopsis thaliana* [31], *Medicago truncatula* [32], and *Glycine max* [33] has revealed their roles in the transport of phenolic compounds. However, despite the pharmacological importance of HPGs, including ACT, the transporters responsible for their import into vacuoles remain largely uncharacterized.

*R. glutinosa*, a medicinal plant belonging to the family Orobanchaceae, is renowned for producing high-value HPGs, with ACT serving as a critical quality control marker due to its potent pharmacological activities [8,9]. Transcriptome-wide analyses have identified 66 MATE family transporters in *R. glutinosa*, which are phylogenetically categorized into distinct subgroups associated with diverse metabolite transport activities, including phenolics and alkaloids [34]. Among these, only two potential vacuolar-localized MATE transporters, RgMATE6 and RgMATE12, were identified. Both contain dual MatE domains characteristic of this transporter family [35] but exhibit critical structural and functional divergences: RgMATE6 comprises 524 amino acids with 12 transmembrane helices, whereas RgMATE12 consists of 512 residues and 9 transmembrane segments [34]. To prioritize candidate transporters for HPGs, this study employed in silico analyses of RgMATE protein features and correlation analysis between HPG accumulation and *RgMATE* expression. These approaches highlighted RgMATE6 as the most promising candidate for mediating vacuolar HPG transport. Given the established role of ACT as a key phenolic-derived HPGs component [8,9], we proposed that RgMATE6 facilitates both ACT transport and its vacuolar accumulation, potentially influencing biosynthesis. To test this hypothesis, a comprehensive functional characterization of RgMATE6 was conducted using integrated in vitro and in vivo assays. By deciphering the specific function underlying RgMATE6-mediated ACT transport, this study establishes a foundational framework for engineering HPG production in medicinal plants.

## 2. Results

### 2.1. Sequence Analysis of the RgMATE Proteins

**Figure 1:**
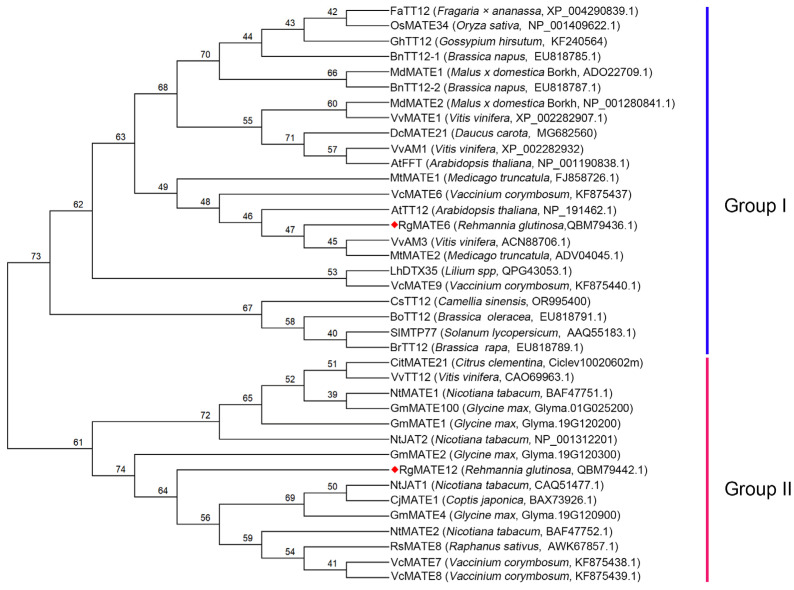
Phylogenetic tree for RgMATE6 and RgMATE12 with other reported plant homologs. The red diamond highlights the RgMATEs investigated in this study.

To analyze the potential functions of the RgMATEs, we reconstructed a phylogenetic tree that included the two RgMATEs and 36 reported vacuolar MATE transporters from diverse plant species. This analysis revealed two evolutionarily distinct clades (Group I and Group II), which correlated with substrate specificity (Figure 1). Group I contained RgMATE6, which clustered closely with phenolic-associated transporters such as VvAM1/VvAM3 from *Vitis vinifera*, AtTT12 from *A. thaliana*, and MtMATE2 from *Medicago truncatula*. Group II included RgMATE12, which grouped with transporters specialized in alkaloid and terpenoid sequestration, including NtJAT2 from *Nicotiana tabacum*, CjMATE1 from *Coptis japonica*, and GmMATE2 from *Glycine max*. Additionally, a multiple sequence alignment of RgMATE6 and RgMATE12 with four vacuolar MATE transporters involved in phenolic compound transport showed that RgMATE6 shared approximately 50% sequence identity with these phenolic transporters, whereas RgMATE12 exhibited only ~30% identity to the reference sequences (Figure S1). These results suggest that RgMATE6 likely retains ancestral activity in vacuolar phenolic transport, consistent with the roles of its Group I homologs, whereas RgMATE12 may have evolved distinct substrate preferences, potentially transporting alkaloids and terpenoids, in line with the functional specialization observed in Group II transporters.

### 2.2. Correlation Analysis Between the HPG Accumulation and RgMATE Expression in R. glutinosa

**Figure 2:**
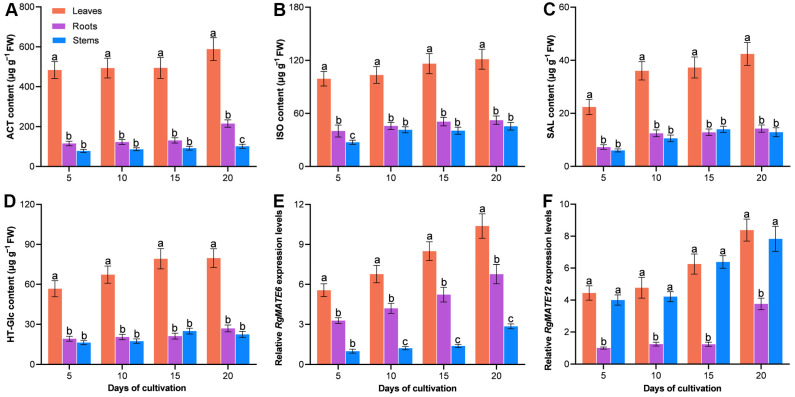
The HPG contents (**A**–**D**) and the expression levels of *RgMATE*6 (**E**) and *RgMATE12* (**F**) in different tissues of *R*. *glutinosa* across cultivation stages. The error bars represent the standard deviations of the mean (*n* = 3, *p* < 0.05). Lowercase letters indicate significant differences at the 0.05 level among different groups (the same convention applies below).

To investigate the correlation between the HPG accumulation and *RgMATE* expression in *R. glutinosa*, the contents of four major HPGs (ACT, isoacteoside (ISO), salidroside (SAL), and hydroxytyrosol-O-glucoside (HT-Glc)) were quantified, and the tissue-specific expression profiling of *RgMATE6* and *RgMATE12* (in roots, stems, and leaves) was analyzed across cultivation stages. The quantification of the four HPGs relied on external calibration curves (Figure S2), which showed excellent linear relationships with coefficients of determination (R^2^) greater than 0.995. As shown in Figure 2, the HPG accumulation exhibited significant variation among tissues: leaves consistently demonstrated the highest levels, followed by roots, while stems showed minimal accumulation. For example, at 20 days of cultivation, the HPG contents in leaves were 5.81-fold (ACT), 2.66-fold (ISO), 3.29-fold (SAL), and 3.54-fold (HT-Glc) higher than those in stems. Representative HPLC chromatograms confirming the separation and detection of these four HPGs in different tissues are provided in Figure S3. Notably, ACT was the most predominant HPG in leaves during the entire cultivation period. At 20 days, for instance, ACT levels in leaves were 4.85-, 13.89-, and 7.38-fold higher than those of ISO, SAL, and HT-Glc, respectively. The expression of *RgMATE6* exhibited a similar tissue-specific pattern to the HPG accumulation, with leaves showing the highest expression and stems showing the lowest. At 20 days of cultivation, *RgMATE6* expression levels in leaves were 1.54- and 3.64-fold higher than those in roots and stems, respectively. In contrast, RgMATE12 exhibited a divergent pattern, with both leaves and stems displaying higher expression levels than roots, and no significant difference was observed between leaves and stems. For example, at 20 days of cultivation, the *RgMATE12* expression levels in leaves and stems were 2.23-fold and 2.02-fold higher than those in roots, respectively.

These results indicated a strong positive Pearson correlation coefficient (*r* > 0.83) between the HPG contents and *RgMATE6* expression levels across tissues and cultivation stages (Table 1), as visually represented in the scatter plot of Figure S4, with ACT content demonstrating the strongest correlation (*r* = 0.861). Conversely, HPG contents exhibited a weaker correlation (*r* < 0.60) with *RgMATE12* expression levels (Table 1). These findings suggest that RgMATE6 is the superior candidate for involvement in HPG transport, particularly for ACT.

**Figure 3:**
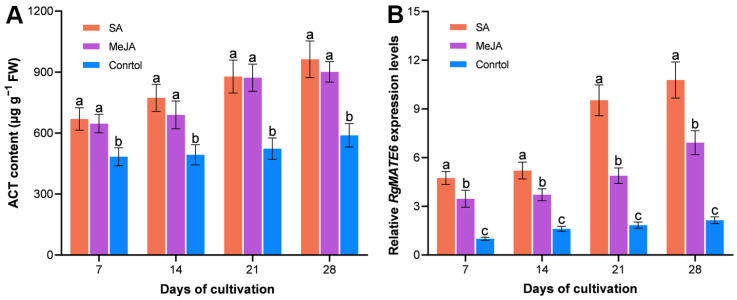
The ACT contents (**A**) and the *RgMATE6* expression levels (**B**) in *R. glutinosa* under SA and MeJA treatments across cultivation stages. Lowercase letters indicate significant differences at the 0.05 level among different groups.

Given the evidence from previous studies that salicylic acid (SA) and methyl jasmonate (MeJA) as elicitors enhance ACT production [8,36], further investigations were conducted to study the effect of SA or MeJA treatment on the content of ACT and *RgMATE6* expression levels in leaves. The treatment of leaves resulted in significant differences in ACT accumulation compared to controls, with SA-treated leaves having the highest ACT levels, followed by MeJA-treated leaves. Following the 28-day treatment period, the content of ACT in SA- and MeJA-treated leaves was found to be 1.64- and 1.53-fold higher than in the control group, respectively. A similar trend was observed in *RgMATE6* expression levels, with SA- and MeJA-treated leaves exhibiting approximately 5- and 3-fold higher expression levels compared to the control group after 28 days of treatment (Figure 3 and Figure S5). The results indicated a strong correlation between ACT accumulation and *RgMATE6* expression levels (*r* > 0.86 across treatments) (Table S1).

Collectively, these findings suggest that RgMATE6 could be a key transporter of HPGs in *R. glutinosa*, with its expression level strongly coupled to both tissue-specific and phytohormone-induced ACT accumulation.

### 2.3. RgMATE6 Transports ACT More Efficiently than Other HPGs in the Nicotiana benthamiana System

**Figure 4:**
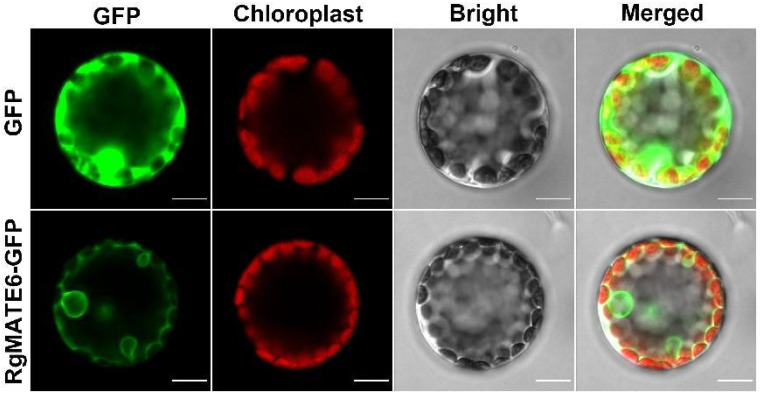
Localization and uptake assays of RgMATE6 in *N. benthamiana*. Subcellular localization of RgMATE6 in *N. benthamiana* leaf mesophyll protoplasts (left to right): GFP signal, chloroplast autofluorescence, bright-field view, and merged image. The green fluorescence represents the GFP-tagged RgMATE6 protein, and the red indicates chlorophyll autofluorescence (scale bars = 10 μm).

**Figure 5:**
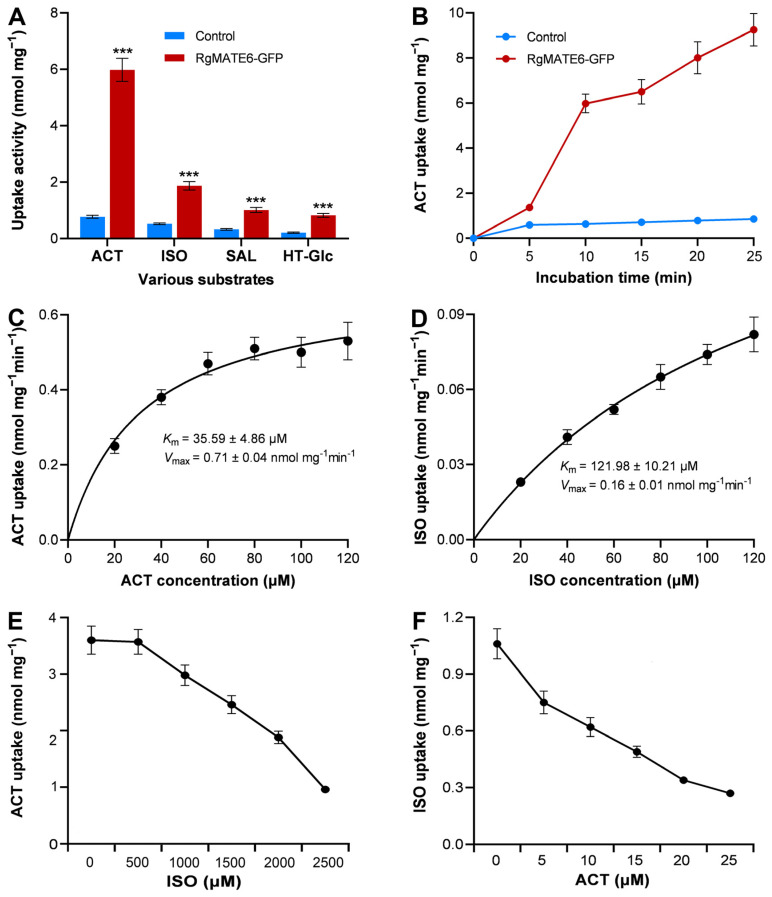
Uptake assays of ACT and the other three HPG by vacuolar membrane vesicles from *N*. *benthamiana* expressing *RgMATE6-GFP* or control. (**A**) Uptake activities of *RgMATE6-GFP* toward various substrates; (**B**) time-dependent uptake of ACT into vesicles from *N. benthamiana* expressing *RgMATE6-GFP*; (**C**) concentration-dependent uptake of ACT into vesicles from *N. benthamiana* expressing *RgMATE6-GFP*; (**D**) concentration-dependent uptake of ISO into the vesicles from *N. benthamiana* expressing *RgMATE6-GFP*; (**E**) inhibition of ACT uptake into vesicles from *N. benthamiana* expressing *RgMATE6-GFP* by ISO; (**F**) inhibition of ISO uptake into the vesicles from *N. benthamiana* expressing *RgMATE6-GFP* by ACT. Asterisks “***” indicate statistically significant differences at the 0.001 level, respectively.

To deepen our understanding of the substrate specificity and preferred substrates of RgMATE6, the constructs pBI121-RgMATE6-GFP (Figure S6A) and the control vector pBI121-GFP (control) were transiently expressed in protoplasts isolated from the mesophyll cells of *N. benthamiana*. A distinct GFP fluorescence signal was observed in protoplasts expressing *RgMATE6-GFP*, localizing to the vacuolar membrane. In contrast, the control GFP signal was predominantly detected within the cytosol (Figure 4). These findings provide unequivocal confirmation of the successful expression and precise localization of the RgMATE6-GFP protein at the vacuolar membrane in plant cells, as previously mentioned [34]. Subsequently, the uptake activities from the vacuole membrane vesicles expressing *RgMATE6-GFP* and *GFP* (control) were analyzed using the four HPG compounds after incubation for 20 min. A notable enhancement in uptake activity was observed for all four HPGs in vacuole membrane vesicles expressing *RgMATE6-GFP*, in comparison to the control (Figure 5A). Specifically, ACT displayed the highest uptake activity, followed by ISO and SAL, whereas the uptake of HT-Glc by RgMATE6-GFP exhibited the lowest activity. The time-course analysis showed a significant increase in the HPG uptake amounts from the vacuole membrane vesicles expressing *RgMATE6*-GFP compared to the control (Figure 5B). Kinetic analyses further revealed a *K*_m_ of 35.59 μM for ACT uptake, alongside a *V*_max_ of 0.71 nmol mg^−1^ min^−1^. Additionally, the uptake of ISO by RgMATE6-GFP exhibited a *K*_m_ of 121.98 μM and a *V*_max_ of 0.16 nmol mg^−1^ min^−1^ (Figure 5C,D). These results indicate that RgMATE6 possesses distinct transport activities towards the tested HPGs, with optimal efficiency observed for ACT.

To further explore the substrate preferences of RgMATE6, a competition assay was performed. These data revealed that ISO inhibited the uptake of ACT only at the higher concentrations (Figure 5E), whereas ACT impeded the uptake of ISO at the lower concentrations (Figure 5F). These results suggested a notable competitive interaction between the import of ISO and ACT. These findings provide compelling evidence that RgMATE6 transports ACT more efficiently than the other three HPGs tested.

### 2.4. RgMATE6 Facilitates the Transport of ACT into Vacuoles in R. glutinosa

**Figure 6:**
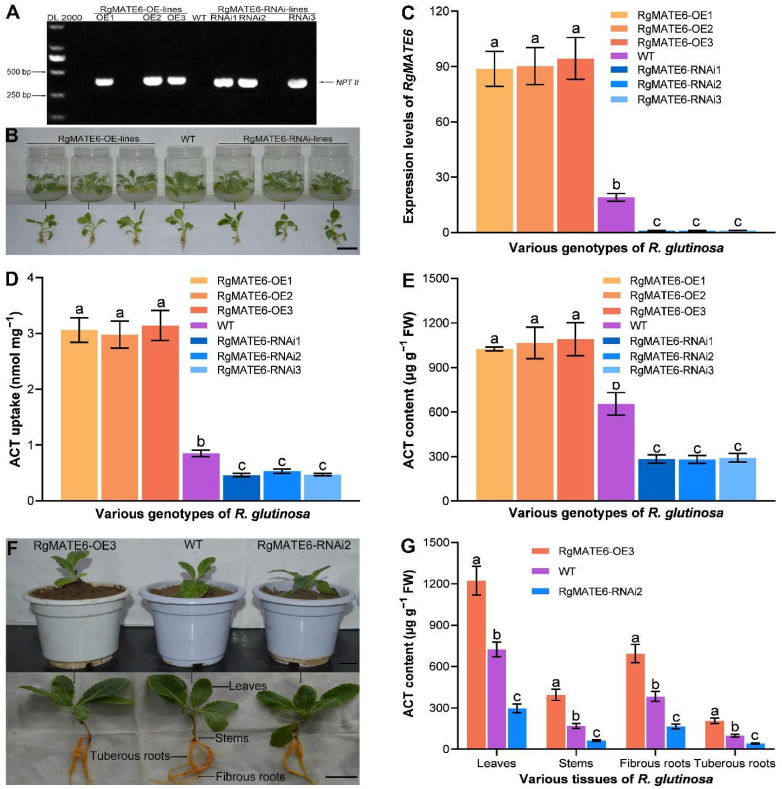
Confirmation of positive *RgMATE6* transgenic *R. glutinosa* and assay of ACT content in these transgenic lines. (**A**) Agarose gel electrophoresis images showing *NPTII* PCR products amplified from transgenic *RgMATE6* and WT plants; (**B**) photographs of the sterile seedlings from the transgenic and WT *R. glutinosa* after 30 days of cultivation (scale bar = 4 cm); (**C**) the relative expression levels of *RgMATE6* in the transgenic and WT *R. glutinosa*; (**D**) uptake assay of ACT into vacuole membrane vesicles from different genotype *R. glutinosa*; (**E**) ACT content assay from different genotype *R. glutinosa*; (**F**) photographs of various genotype plants cultured in pots filled with soil for 30 days (scale bar = 4 cm); (**G**) ACT content assay in various tissues from these plants. Lowercase letters indicate significant differences at the 0.05 level among different groups.

To investigate the molecular function of RgMATE6 in *R. glutinosa*, we generated *RgMATE6*-overexpressing (RgMATE6-OE) and RgMATE6-RNAi transgenic lines using the CaMV35S promoter for gene overexpression and repression, respectively (Figure S6B,C). Successful generation of these transgenic lines was confirmed (Figure 6 and Figure S7A–H). PCR analysis of transgenic shoots and wild-type (WT) plants detected a 385 bp fragment of the *NPTII* gene (conferring kanamycin resistance) exclusively in the transgenic lines, confirming their genetic modification (Figure 6A). These lines were then propagated clonally via shoot culture. After 30 days of sterile cultivation, we performed qRT-PCR analysis on the leaves (Figure 6B). The results showed significantly higher *RgMATE6* expression in RgMATE6-OE lines and reduced expression in RgMATE6-RNAi lines compared to the WT control (Figure 6C).

To validate the transport activity of RgMATE6 in *R. glutinosa*, vacuolar membrane vesicles isolated from the transgenic and WT seedling leaves were incubated with ACT for 20 min. As shown in Figure 6D, the ACT uptake amount in vesicles from RgMATE6-OE lines was significantly higher than that in WT controls. For example, RgMATE6-OE3 exhibited a 3.7-fold increase in ACT uptake amount compared to WT. Conversely, the vesicles from RgMATE6-RNAi lines showed markedly reduced uptake activity. For example, RgMATE6-RNAi2 displayed only 62% of the uptake levels of WT. These results demonstrate that *RgMATE6* overexpression enhances ACT vacuolar sequestration, while its repression suppresses transport efficiency in *R. glutinosa*.

### 2.5. Genetic Manipulation of RgMATE6 Modifies ACT Production in R. glutinosa by Facilitating Vacuolar Import

To assess the function of RgMATE6 in vivo, the ACT content was quantified in the leaves of transgenic *R. glutinosa* seedlings (Figure 6E). The RgMATE6-OE lines showed a significant increase in ACT content compared to the WT control. For example, the ACT content in the leaves of RgMATE6-OE3 was approximately 1.7-fold higher than that of the WT. Conversely, the RgMATE6-RNAi lines exhibited a significant decrease in ACT content compared to the WT. For instance, the ACT content in the seedlings of RgMATE6-RNAi2 lines was approximately 50% of that in the WT. These results demonstrate that overexpression of *RgMATE6* enhances ACT accumulation, while its repression diminishes it.

To validate these findings, we cultivated the RgMATE6-OE3 and RgMATE6-RNAi2 lines in pots filled with soil for 30 days, followed by ACT quantification across various tissues. Overall, the RgMATE6-OE3 plants showed significantly higher ACT levels in all tissues compared to the WT plants (Figure 6F,G). For example, the leaves of RgMATE6-OE3 exhibited approximately 1.7-fold higher ACT content, while the tuberous roots showed a 2.1-fold increase relative to the WT. In contrast, the RgMATE6-RNAi2 plants exhibited significantly lower ACT content. The leaves and tuberous roots exhibited approximately 40% of the WT levels. These data confirm RgMATE6’s role in ACT accumulation and reveal the tissue-specific regulatory variation.

### 2.6. RgMATE6 Shows Positive Coordination with the Expression of ACT Biosynthetic Genes in R. glutinosa

**Figure 7:**
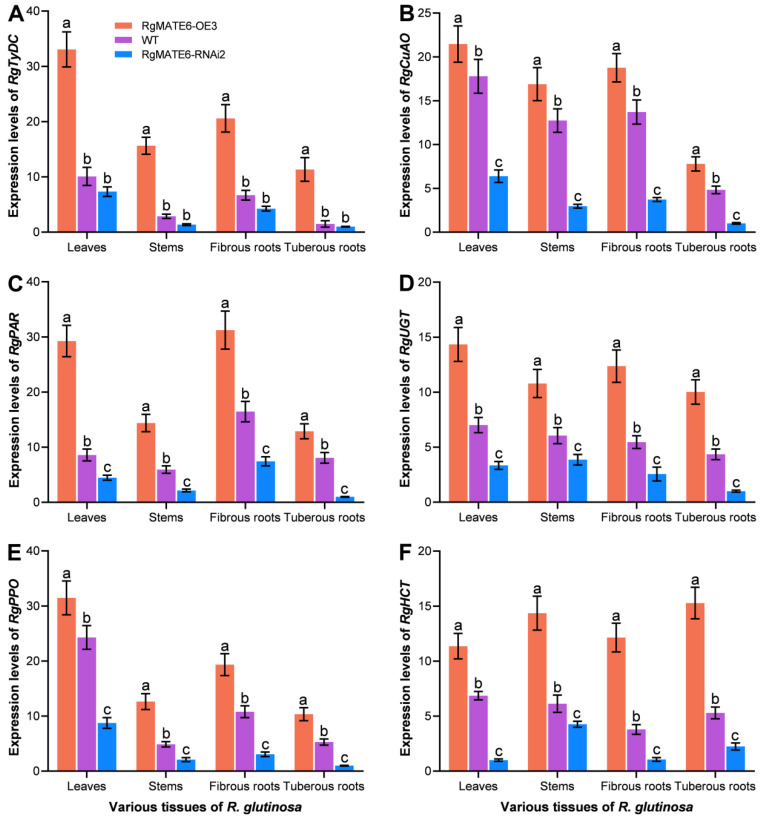
Expression profiles of six genes involved in ACT biosynthesis in various tissues of the transgenic and WT *R. glutinosa*. (**A**) *RgTyDC*; (**B**) *RgCuAO*; (**C**) *RgPAR*; (**D**) *RgUGT*; (**E**) *RgPPO*; (**F**) *RgHCT.* Lowercase letters indicate significant differences at the 0.05 level among different samples.

To examine potential relationships between RgMATE6 and ACT biosynthesis, we compared tissue-specific expression profiles of six key ACT biosynthetic genes (RgTyDC encoding tyrosine decarboxylase, RgCuAO copper amine oxidase, RgPAR phenylacetaldehyde reductase, RgPPO polyphenol oxidase, RgUGT UDP-glucose glucosyltransferase, and RgHCT shikimate O-hydroxycinnamoyltransferase) [8,20] across three *R. glutinosa* genotypes at 30 days of cultivation. RgMATE6-OE3 plants showed significantly elevated expression of all six genes across tissues compared to WT controls, while RgMATE6-RNAi2 plants exhibited substantially reduced expression levels in corresponding tissues (Figure 7). For example, *RgPAR* expression in RgMATE6-OE3 leaves and fibrous roots increased by 3.5-fold and 1.9-fold, respectively, relative to WT. *RgHCT* expression displayed a similar pattern with approximately 1.7-fold upregulation in RgMATE6-OE3 plants. In contrast, *RgPAR* expression in RgMATE6-RNAi2 leaves and fibrous roots decreased to 50% of WT levels, with *RgHCT* expression reduced to 70% of WT levels. These results reveal coordinated expression patterns between *RgMATE6* and ACT biosynthetic genes in *R. glutinosa*.

## 3. Discussion

In *R. glutinosa*, ACT, a pivotal HPG, manifests well-documented bioactivities [8,9]. Consequently, enhancing ACT biosynthesis in this medicinal plant holds significant agricultural and pharmacological value. Building on a previous study that identified RgMATE6 and RgMATE12 as potential vacuolar transporters in *R. glutinosa* [34], our phylogenetic and homology analyses revealed that RgMATE6 shares closer evolutionary relationships and higher sequence identity with VvAM3 (an anthocyanin transporter from *V. vinifera*) [37] and MtMATE2 (a flavonoid glycoside transporter from *M. truncatula*) [32] compared to RgMATE12. These findings suggest that RgMATE6 may function in phenolic transport. In contrast, RgMATE12 shows homology to transporters of non-phenolic metabolites, such as NtJAT2 (an alkaloid transporter from *N. tabacum*) [38], CjMATE1 (an alkaloid transporter from *Coptis japonica*) [39], and GmMATE1 (a terpenoid transporter from *Glycine max*) [33], implying potential roles in alkaloid or terpenoid transport. Previous studies have reported that the production of plant-specialized metabolites shows a positive correlation with transcript levels of genes encoding MATE transporter proteins responsible for their vacuolar sequestration [25,31]. In this work, tissue-specific correlation analysis demonstrated a stronger positive association between the HPG accumulation and *RgMATE6* expression than with *RgMATE12* expression. Furthermore, treatment with metabolic elicitors SA and MeJA [40] produced a pronounced elevation in ACT production and the upregulation of *RgMATE6* expression. Collectively, these results imply that RgMATE6 may contribute to ACT transport and vacuolar accumulation in *R. glutinosa*, though direct functional validation remains required.

The transformation of mesophyll cells from *N. benthamiana* has been commonly used as a functional expression system for plant vacuolar transporters in previous studies [23,41]. To characterize RgMATE6’s substrate specificity/preference, *RgMATE6-GFP* was expressed in *N. benthamiana* cells, which confirmed vacuolar membrane localization. Transport assays using vacuolar membrane vesicles demonstrated RgMATE6’s capacity to mediate HPG transport, with the highest efficiency for ACT. Competitive uptake experiments between ISO and ACT further confirmed RgMATE6’s preferential affinity for ACT. Most importantly, ACT uptake assays using vacuolar membrane vesicles from transgenic *R. glutinosa* plants verified RgMATE6’s essential role in ACT transport. Collectively, these results from *N. benthamiana*, and *R. glutinosa* systems consistently demonstrate that RgMATE6 functions to efficiently transport ACT into vacuoles at the molecular level.

Plant vacuoles are well-known as central storage compartments for crucial secondary metabolites within plant cells [23,24,25]. Having demonstrated through in vitro assays that RgMATE6 facilitates the import of ACT into the vacuoles, we next sought to elucidate its molecular function in vivo. We found that overexpression of *RgMATE6* enhanced ACT accumulation, while suppression of *RgMATE6* reduced accumulation in *R. glutinosa* seedlings and various tissues. These in vivo results not only confirmed our in vitro findings but also revealed tissue-specific accumulation patterns. This demonstrates that RgMATE6 positively regulates ACT production in *R. glutinosa*. MATE transporters typically mediate metabolite transport across the tonoplast into the vacuolar lumen [29,31]. Our results provide compelling evidence that RgMATE6 serves as a principal transporter governing ACT accumulation in *R. glutinosa*.

**Figure 8:**
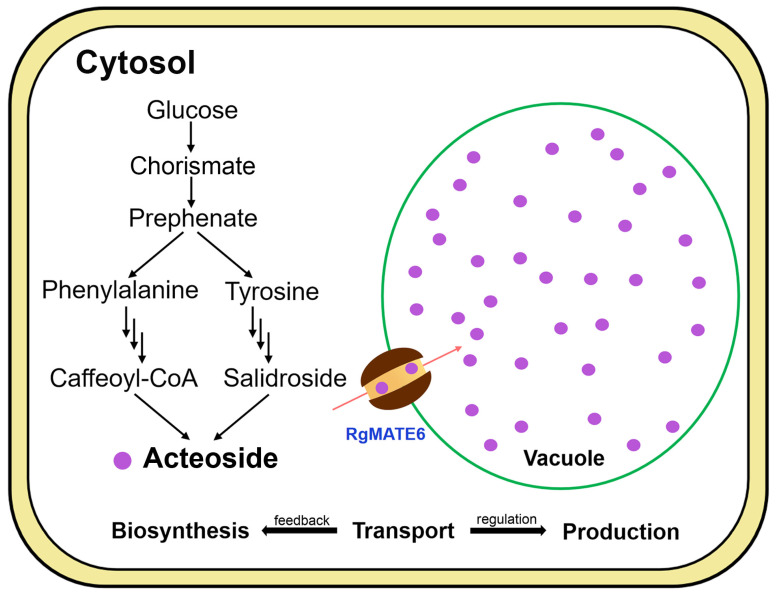
Function model of RgMATE6 in the transport and production of ACT in *R. glutinosa*. The multiple arrows indicate the metabolism of cinnamic acid, *p*-coumaric acid, and caffeic acid in the phenylpropanoid pathway, as well as tyramine and tyrosol in the tyrosine metabolism pathway.

MATE transporters have been widely demonstrated to act as key regulators of secondary metabolite biosynthesis in plants [29,30,32]. Our study demonstrated that *RgMATE6* overexpression upregulated ACT biosynthetic genes, whereas its repression downregulated their expression, implying its potential association with the ACT biosynthetic pathway. The coordinated processes of secondary metabolite biosynthesis, transport, and accumulation involve MATE transporters in feedback regulation mechanisms in plants [24,31,42]. In this study, *RgMATE6* was shown to positively coordinate the expression of the ACT biosynthetic genes, speculating its critical role in tuning a positive feedback that may enhance ACT biosynthesis. Furthermore, RgMATE6-mediated transport of ACT into the vacuole highlights the integrated regulation of these processes for optimal metabolite production, as previously reported [24,25,27,33]. Thus, *RgMATE6* enhances ACT production by both promoting vacuolar accumulation and possibly synergizing with the biosynthetic pathway (Figure 8). These findings provide novel insights into the ACT production mechanisms and potential strategies for enhancing valuable HPG production through genetic or biotechnological approaches.

ACT represents a key example among the diverse HPGs produced by plants. However, the functional characteristics of other MATE transporters involved in HPGs transport remain incompletely understood. Therefore, additional studies are needed to identify other transporters mediating HPG import, as this would significantly advance our understanding of the regulatory networks controlling the HPG biosynthesis in plants. Investigating alternative transport systems may lead to more efficient approaches for utilizing plant transporter resources, potentially enabling the development of novel methods and targeted strategies to enhance the production of both ACT and other valuable HPGs in plants.

## 4. Materials and Methods

### 4.1. In Silico Analysis

For phylogenetic analysis, RgMATE6 (GenBank ID: QBM79436.1) and RgMATE12 (QBM79442.1), along with other reported plant MATE proteins, were retrieved through BLASTP searches against the NCBI database (https://blast.ncbi.nlm.nih.gov/Blast.cgi, accessed on 16 November 2025). Redundant sequences were removed using CD-HIT [43] with a 90% identity cutoff. From the retained unique sequences, functionally characterized plant vacuolar MATE proteins involved in metabolite transport were selected through manual curation. Sequence alignment was performed using ClustalW in MEGA software package, version 12.0 [44,45], with gap opening and extension penalties set to 10.00 and 0.10 for both pairwise and multiple alignments. Gappy or poorly aligned regions were manually inspected and trimmed to improve alignment quality. Phylogenetic reconstruction was conducted using the maximum likelihood method in MEGA12 with the Jones–Taylor–Thornton (JTT) model and 1000 bootstrap replicates. Additionally, multiple sequence alignment for RgMATE protein identity analysis was performed using DNAMAN 7.0 software.

### 4.2. Cultivation of R. glutinosa, RNA Extraction, and cDNA Synthesis

The seedlings of *R. glutinosa* cultivar ‘Wen 85-5’ were aseptically propagated in glass tubes containing 50 mL of solidified Murashige and Skoog (MS) medium (Solarbio, Beijing, China), with 3% sucrose supplementation. The seedlings were grown in a controlled-environment greenhouse (26 °C, 14 h light/10 h dark) photoperiod until reaching the six-leaf developmental stage. Subsequently, the young plants were moved to pots containing organic nutrient-rich soil and cultivated in the greenhouse at 65% relative humidity. For the purpose of conducting a tissue-specific analysis, roots, stems and leaves of the plants were harvested at 5, 10, 15, and 20 days of cultivation, respectively. For elicitor treatments, the plants at 10 days post-transplantation were irrigated every four days with 100 mL of water (control), 20 μM SA, or 20 μM MeJA, respectively. Leaf samples from each treatment group were collected at 7, 14, 21, and 28 days after treatment initiation (Figure S5). Each experimental group comprised three biological replicates.

To ensure high-quality RNA extraction, all samples were immediately flash-frozen in liquid nitrogen. The total RNA was subsequently isolated employing TRIzol reagent (Invitrogen, Carlsbad, CA, USA), following the manufacturer’s instructions. The RNA yield and quality were measured with a NanoDrop 2000 system (Thermo Scientific, Wilmington, DE, USA). RNA integrity was checked by 1% agarose gel electrophoresis. cDNA was generated from 1 μg RNA using HiScript III Reverse Transcriptase (Vazyme, Nanjing, China) and oligo-dT primers. Subsequent to this, the synthesized cDNA was diluted 1:5 (*v*/*v*) for downstream applications.

### 4.3. Quantitative Real-Time PCR Analysis

Gene-specific primers were computationally designed (Beacon Designer), with sequences provided in Table S2. Gene expression was quantified by quantitative real-time PCR (qRT-PCR) with SYBR Green Master Mix (Fermentas) using a Bio-Rad iQ5 system, in accordance with established protocols [46]. Relative gene expression levels were calculated using *RgActin* as the internal control. The 2^−ΔΔCT^ algorithm [47] was employed for gene expression analysis, incorporating triplicate biological and technical replicates.

### 4.4. Measurement of the HPGs in R. glutinosa

The contents of the HPGs, including ACT, ISO, SAL, and HT-Glc (MedChemExpress, Shanghai, China; HPLC-determined purity >98%), were quantified using the following protocol: 0.5 g of fresh sample was ground into a fine powder and then extracted with 10 mL of methanol for 5 h at room temperature. To enhance the extraction process, sonication in an ice bath was employed for a cumulative time of 5 min, with intermittent sonication cycles of 10 s on and 5 s off. Following 12,000× *g* centrifugation, the collected pellet was lyophilized and redissolved in 5 mL of 30% (*v*/*v*) aqueous acetonitrile. The sample was passed through a 0.22-μm sterile filter for purification before HPLC measurement. HPG content was determined using an Agilent 1260 series HPLC instrument (Agilent Technologies, Santa Clara, CA, USA). A series of standard solutions at seven different concentrations was prepared by diluting the certified reference materials for each analyte (ACT, ISO, SAL, and HT-Glc). The concentration ranges were 0–32 mg L^−1^ for ACT and ISO and 0–15 mg L^−1^ for SAL and HT-Glc. Each calibration curve was constructed by plotting the peak area against the corresponding concentration, and all showed excellent linearity with a coefficient of determination greater than 0.995. The mobile phase for ACT consisted of 30% acetonitrile and 70% water with 0.1% phosphoric acid. For both ISO and SAL analyses, the mobile phase contained 20% pure acetonitrile mixed with 80% water with 0.1% phosphoric acid. HT-Glc separation employed a mobile phase formulation of 33% acetonitrile combined with 67% water with 0.1% phosphoric acid. The chromatographic separation was performed at a constant flow rate of 0.8 mL per minute. For analyte detection, different wavelengths were optimized for each compound: 330 nm for ACT, 334 nm for ISO, 275 nm for SAL, and 280 nm for HT-Glc, while keeping the analytical column at 30 °C throughout the analysis. Quantitative determination of the HPG levels, expressed in micrograms per gram of fresh weight, was achieved through external calibration with certified reference materials. Each experimental group included triplicate biological samples.

### 4.5. Construction of Various Expression Vectors

For *RgMATE6* constructs, the gene fragments, including complete and truncated open reading frames (ORFs), were PCR-amplified employing Phanta MaX Super-Fidelity DNA Polymerase (Vazyme, Nanjing, China) with specific primer pairs detailed in Table S3. For transient expression in *N. benthamiana*, the full-length *RgMATE6* ORF was ligated into the *Xho*I site of the pBI121-GFP vector (Miaoling Biology, Wuhan, China), producing a CaMV35S promoter-driven C-terminal GFP fusion construct (pBI121-RgMATE6-GFP; Figure S6A). The full-length RgMATE6 coding sequence was directionally cloned into pBI121-GFP (Miaoling, Wuhan, China) using *Kpn*I/*Xho*I restriction sites, generating a CaMV35S-driven overexpression vector containing the *NPTII* kanamycin resistance gene. (RgMATE6-OE; Figure S6B). For RNAi vector construction, a partial *RgMATE6* ORF fragment was cloned into the *Bsa*I site of pRNAi-GG (Biovector Science Lab, Beijing, China), generating the RgMATE6-RNAi construct with CaMV35S promoter and *NPTII* selection marker (Figure S6C). All recombinant plasmids were introduced into *Escherichia coli* DH5α, with correct clones identified by DNA sequencing (Sangon, Shanghai, China).

### 4.6. N. benthamiana Transformation and Vacuolar Membrane Vesicle Preparation

Leaf mesophyll protoplasts were extracted from six-leaf stage *N. benthamiana* plants following an adapted version of the *Arabidopsis* protoplast isolation method [48]. The pBI121-RgMATE6-GFP and pBI121-GFP (control) constructs were transiently transformed into protoplasts via polyethylene glycol-mediated transfection. After 36–48 h incubation at 25 °C, RgMATE6 subcellular localization was analyzed by confocal microscopy (Olympus Corporation, Tokyo, Japan), as aforementioned.

Subsequently, vacuolar membrane vesicles were prepared from the transformed protoplasts following a modified protocol described by Yamanishi and Kasamo [49]. Briefly, the protoplast suspension was prepared in homogenization buffer composed of 0.1 M mannitol, 1.5 mM MES at pH 5.6, 154 mM NaCl, 125 mM CaCl_2_, 5 mM KCl, and 5 mM glucose. Following centrifugation (50,000× *g*, 30 min, 4 °C), the microsomal pellet was resuspended in 36 mL sucrose buffer (0.6 M sucrose, 10 mM KCl, 1 mM EDTA, 2 mM DTT) and layered with 3.5 mL sorbitol buffer (0.25 M sorbitol, 5 mM HEPES-Tris pH 7.5, 1 mM EDTA, 2 mM DTT). After high-speed centrifugation (100,000× *g*, 40 min), the vacuolar membrane interface was collected and preserved in storage solution (0.3 M sorbitol, 0.1 M KCl, 5 mM Tris-MES pH 7.5, 1 mM DTT with protease inhibitors) at −80 °C.

### 4.7. Generation of RgMATE6 Transgenic Lines of R. glutinosa and the Isolation of Vacuolar Membrane Vesicles

Both RgMATE6-OE and RgMATE6-RNAi vectors were delivered into *Agrobacterium tumefaciens* GV3101 through freeze–thaw transformation [50]. For the genetic modulation of *RgMATE6* expression, surface-sterilized leaf explants from *R. glutinosa* seedlings were infected with the engineered *Agrobacterium* strains following established protocols [51]. To verify transgenic integration, putative transgenic shoots (originating from distinct explants subjected to independent *Agrobacterium* co-cultivation steps) were generated on the MS basal medium (Solarbio, Beijing, China) supplemented with additions of 30 g L^−1^ sucrose, 0.5 mg L^−1^ NAA, 3 mg L^−1^ 6-BA, 100 mg L^−1^ kanamycin, 200 mg L^−1^ timentin, and 7.8 g L^−1^ agar. The cultures were maintained under the controlled greenhouse conditions as mentioned above. Genomic DNA was extracted from both transgenic shoots and WT controls employing the CTAB isolation protocol [52]. Transgenic plants were validated by NPTII marker amplification. For further analysis, each positive transgenic line was propagated aseptically through shoot culture to generate multiple clonal plants. Leaves from these transgenic plants after 30 days of culture were used to analyze *RgMATE6* expression levels by qRT-PCR, measure ACT uptake in vacuolar membrane vesicles as described above, and quantify ACT content by HPLC analysis.

### 4.8. Transport Activity Assays

The transport capacity of vacuolar membrane vesicles was assessed at 25 °C according to an optimized protocol [53]. The reaction system, with a total volume of 500 μL, chilled buffer containing 0.4 M sorbitol and 25 mM Tris-MES (pH 8.0) and 0.1% (*w*/*v*) BSA, and four substrates (each at 100 μM): ACT, ISO, SAL and HT-Glc. Subsequent to the incorporation of vesicle addition (approximately 50 μg of membrane protein) to initiate transport, the mixture was vortexed for 10 s. At predetermined intervals (5, 10, 15, 20, and 25 min), 100 μL aliquots were withdrawn and quenched with 1.0 mL of ice-cold termination buffer (0.4 M sorbitol and 25 mM Tris-MES (pH 8.0)). Reaction suspensions were vacuum-filtered via a 0.22 μm filter (pore size). Thereafter, membrane-trapped analytes were extracted in 1 mL of methanol–water mixture (1:1, *v*/*v*) using orbital shaking (180 rpm, 2 h, 25 °C). The resulting eluates were then analyzed by HPLC, following our established chromatographic parameters. All experimental procedures included three independent replicates.

To determine the transport kinetic parameters, transport assays were conducted in a 500 μL reaction system: substrate at gradient concentrations (20–120 μM), pre-cooled buffer as described above, and vesicle preparation were sequentially added to initiate the reaction. Following an incubation period at 25 °C for 20 min, the reaction mixture was vacuum-filtered through a 0.22 μm membrane filter, followed by quantitative analysis of the HPG transport activity using the aforementioned HPLC method. Kinetic parameters, including *K*_m_ and *V*_max,_ were derived from Lineweaver–Burk plots, with all assays performed in triplicate.

For competition assays using vacuolar membrane vesicles from *N. benthamiana*, ACT or ISO was utilized as the uptake substrate at a concentration of 100 µM, with various concentrations of their corresponding competition substrates. Following an incubation of 20 min at 25 °C, the transported amounts of ACT or ISO were measured using HPLC.

### 4.9. Statistical Analysis

All experimental data were statistically analyzed using the statistical package SPSS v. 20.0 (IBM, NY, USA). Gene expression and HPG content differences in *R. glutinosa* were evaluated by one-way ANOVA. For post hoc analysis, we employed both Fisher’s least significant difference test and Duncan’s multiple range test with a 0.05 significance level to detect group differences. Variable correlations were assessed by Pearson’s method, and both the correlation coefficient and the coefficient of determination are reported (*p* < 0.05). We employed Student’s independent t-tests to compare transport activities between experimental treatments and controls (*p* < 0.01 or 0.001). The error bars depicted in all figures represent standard deviations (SD) calculated from a minimum of three biological or technical replicates (*n* = 3).

## 5. Conclusions

This study provides a comprehensive understanding of the role of RgMATE6 in the transport and biosynthesis of ACT. In silico analysis identified *RgMATE6* as a putative phenolic compound transporter, which was further supported by correlation analyses linking its expression to HPG accumulation in *R. glutinosa*. In vivo assays confirmed *RgMATE6* as an HPG transporter with marked specificity for ACT. Functional characterization revealed that *RgMATE6* not only mediates ACT vacuolar import but might also synergize with its biosynthetic pathway. Additionally, tissue-specific expression profiling of ACT biosynthesis-related genes in transgenic plants suggested that *RgMATE6* positively regulates ACT biosynthesis. Collectively, these findings demonstrate that *RgMATE6* enhances ACT production through vacuolar transport/accumulation and potential coordination with biosynthesis via a feedback loop. This work advances the understanding of HPG regulation in plants and highlights biotechnological opportunities for targeted metabolite production.

## Figures and Tables

**Table 1:** The correlation between *RgMATE6* expression levels and ACT content across various treatments was quantified by the Pearson correlation coefficient (*r*). Note: “*” indicates significance at the 0.05 level.

	Expression Levels	RgMATE6	RgMATE12
Content of HPGs			
ACT		0.861 *	0.459
ISO		0.859 *	0.508
SAL		0.854	0.575
HT-Glc		0.839	0.546

## Data Availability

The data sets generated and analyzed in this study are available from the corresponding author upon reasonable request.

## Supplementary Materials

The following supporting information can be downloaded at: https://www.mdpi.com/article/10.3390/plants14233608/s1, Figure S1: Multiple sequence alignment of RgMATE6 and RgMATE12 with homologs from *Arabidopsis thaliana*, *Vitis vinifera*, *Vaccinium corymbosum* and *Medicago truncatula*; Figure S2: The curves for ACT, ISO, SAL and HT-Glc were established by plotting the peak area against the concentration of the analytical standards. The regression equation, coefficient of determination (R^2^), and linear range for each compound are provided in the graph; Figure S3: HPLC chromatogram samples of HPG contents in the leaves of *R. glutinosa* at 20 days of cultivation; Figure S4: Scatter plot analysis of the correlation between the content of four HPGs and the expression of *RgMATE6*; Figure S5: *R. glutinosa* seedlings at various days after SA and MeJA treatments (scale bar = 4 cm); Figure S6: The constructs for various *RgMATE6* vectors. These include the CaMV35S: RgMATE6-GFP (A), RgMATE6-OE (B) and RgMATE6-RNAi (C) constructs; Figure S7: Generation of transgenic RgMATE6 *R. glutinosa* plants. (A) Co-cultivation of the explants and *Agrobacterium tumefaciens*; (B) and (C) the callus induction/selection medium from the explants-transferred; (D) the adventitious shoots induction/selection medium from the callus; (E) and (F) the transgenic seedlings were generated in the selection medium; (G) and (H) acclimatization to organic and field soils (scale bars = 1 cm); Table S1: The correlation between the *RgMATE6* expression levels and the ACT content of *R. glutinosa* under various treatments was quantified by the Pearson correlation coefficient (r). Note: “*” represent significantly different levels of 0.05, respectively; Table S2: Primer sequences used for qRT-PCR analysis; Table S3: The primer sequences of *RgMATE*6 used to constructs, and the primer sequences of positive screened gene in *R. glutinosa*. Note: the lowercases represent the sequences of these vector adapters.

## Abbreviations

The following abbreviations are used in this manuscript:

HPG	Hydroxytyrosol-type phenylethanol glycoside
*R. glutinosa*	*Rehmannia glutinosa*
*N. benthamiana*	*Nicotiana benthamiana*
ORFs	Open reading frames
ACT	Acteoside
ISO	Isoacteoside
SAL	Salacteoside
HT-Glc	Hydroxytyrosol-O-glucoside
SA	Salicylic acid
MeJA	Methyl jasmonate
*A. thaliana*	*Arabidopsis thaliana*
RgTyDC	Tyrosine decarboxylase
RgCuAO	Copper amine oxidase
RgPAR	Phenylacetaldehyde reductase
RgPPO	Polyphenol oxidase
RgUGT	UDP-glucose glucosyltransferase
RgHCT	Shikimate O-hydroxycinnamoyltransferase
