# Supplementary material for: A Multidrug and Toxic Compound Extrusion Transporter, RgMATE6, Facilitates Vacuolar Transport of Acteoside in Rehmannia glutinosa
Source: Plants (Basel). 2025 Nov 26;14(23):3608. doi: 10.3390/plants14233608 (PMC12694110; doi:10.3390/plants14233608)
Supplement: Supplementary file 1 [file plants-14-03608-s001.zip › plants-3940985-supplementary.pdf]

Supplementary file

A Multidrug and Toxic Compound Extrusion Transporter, RgMATE6, Facilitates Vacuolar Transport of Acteoside in *Rehmannia glutinosa*

Yanhui Yang[1,*], Yuying Li[1], Yuxuan Wang[1], Mingjie Li[2], Zhongyi Zhang[2], Ruifang Li[1], Weiwei Wang[1], Fuxi Shen[1], Mengman Yan[1]

[1]School of Bioengineering, Henan University of Technology, Lianhua Street 100, Zhengzhou High-technology Zone, Henan Province, China, 450001

[2]College of Bee Science and Biomedicine, Fujian Agriculture and Forestry University, Jinshan Road, Cangshan District, Fuzhou, China, 350002

***Corresponding author**
Yanhui Yang, E-mail adress: yyhui2004@haut.edu.cn

This file includes:
Figure S1 to Figure S7
Table S1 to Table S3

Figure S1 Multiple sequence alignment of RgMATE6 and RgMATE12 with homologs from *Arabidopsis thaliana, Vitis vinifera, Vaccinium corymbosum* and *Medicago truncatula.*

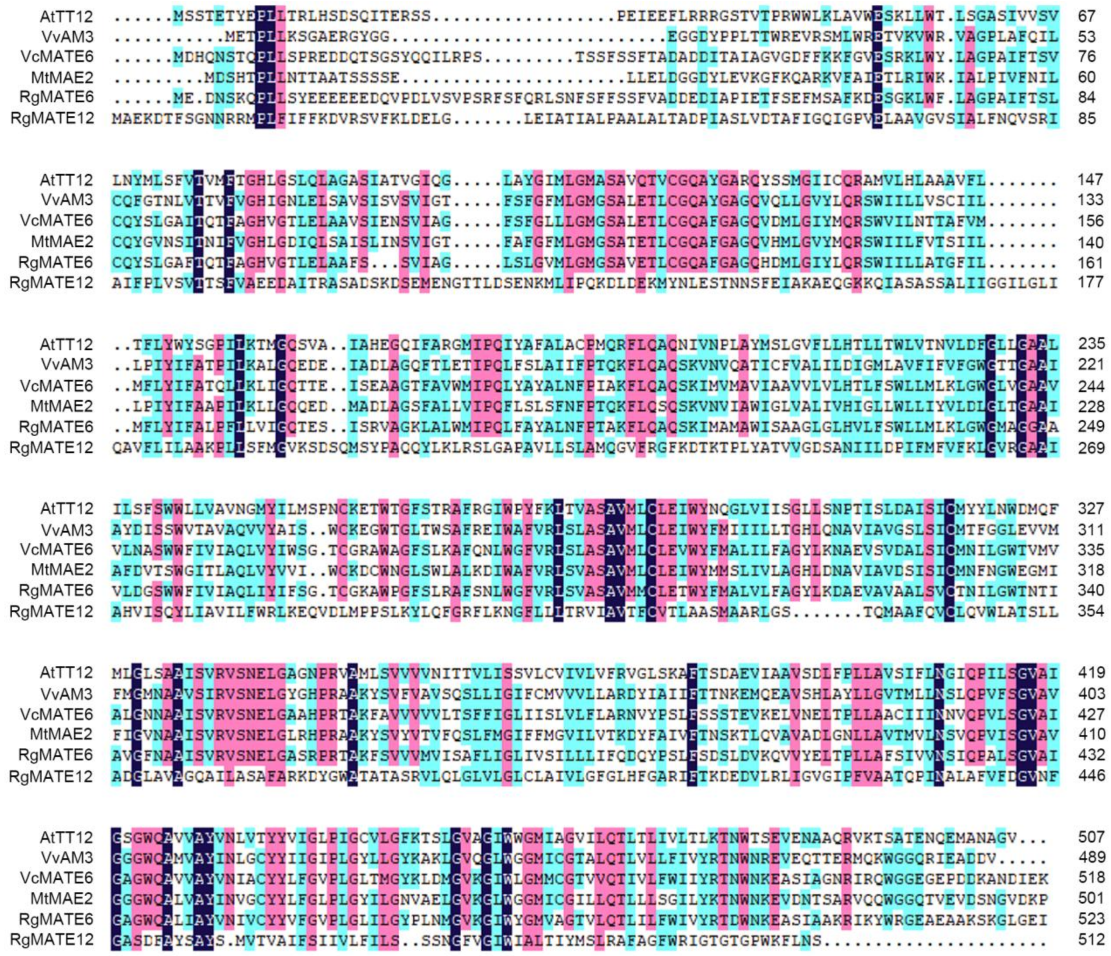

Figure S2 The curves for ACT, ISO, SAL and HT-Glc were established by plotting the peak area against the concentration of the analytical standards. The regression equation, coefficient of determination (R^2), and linear range for each compound are provided in the graph.

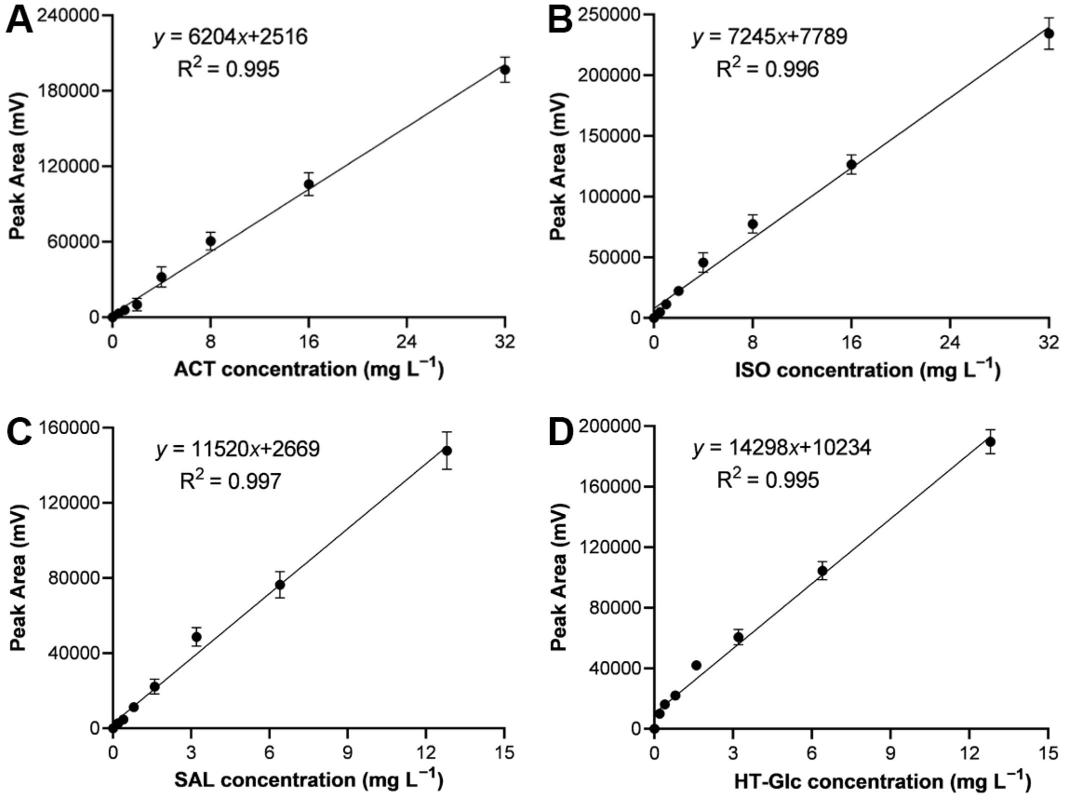

Figure S3 HPLC chromatogram samples of HPG contents in the leaves of *R. glutinosa* at 20 days of cultivation.

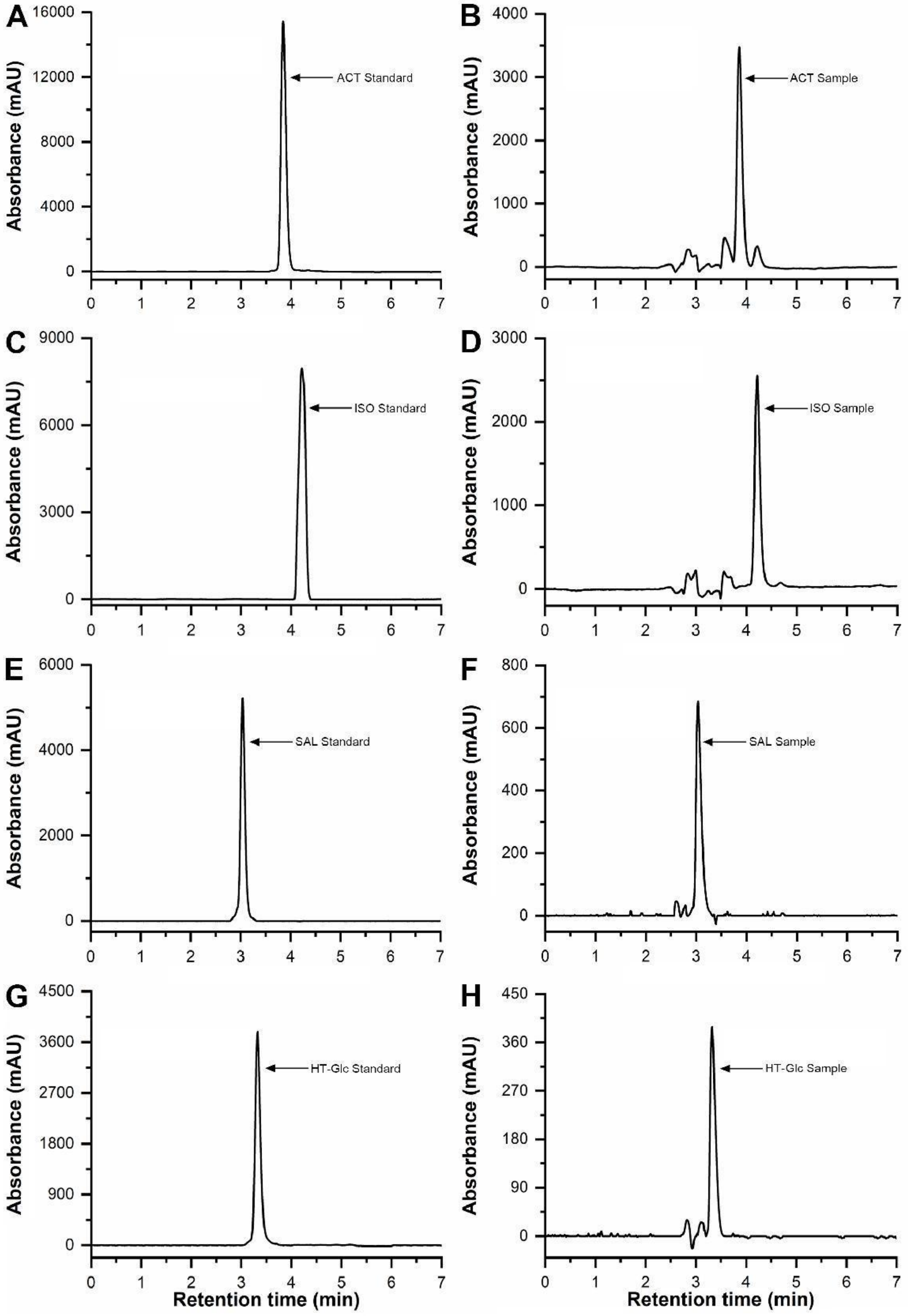

Figure S4 Scatter plot analysis of the correlation between the content of four HPGs and the expression of *RgMATE6*.

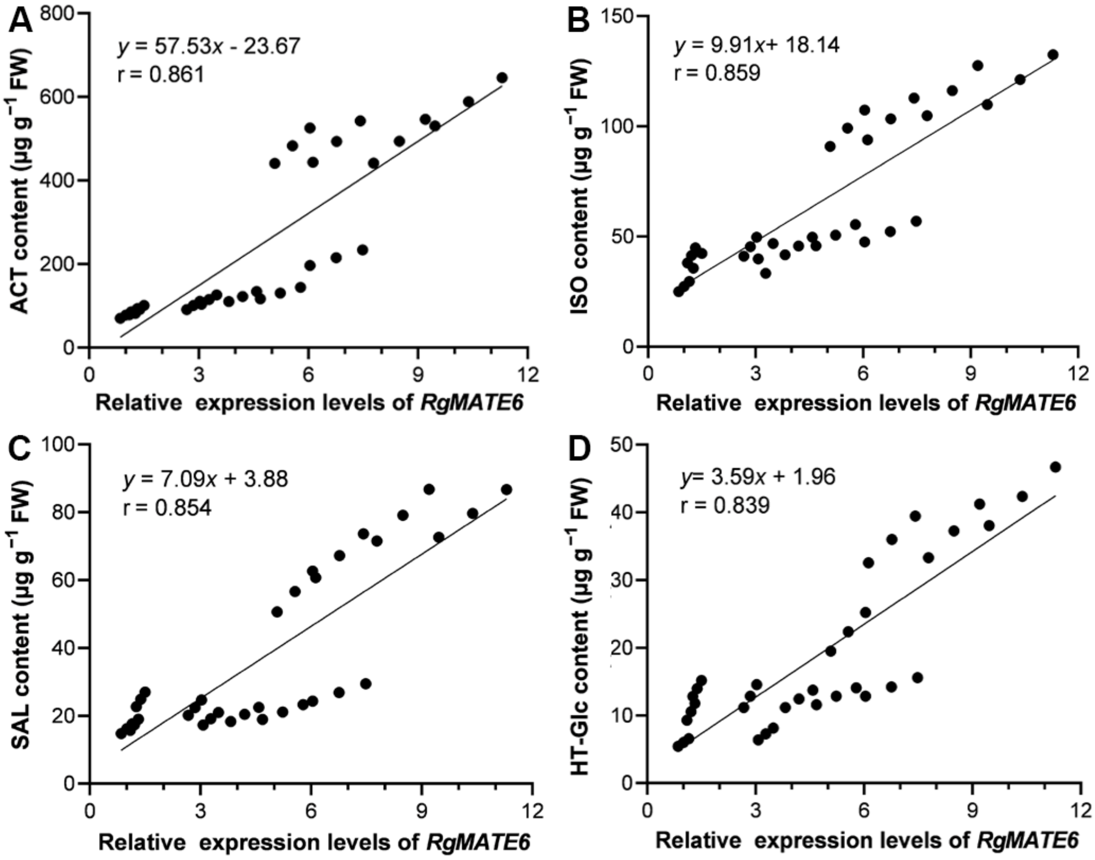

Figure S5 *R. glutinosa* seedlings at various days after SA and MeJA treatments (scale bar = 4 cm).

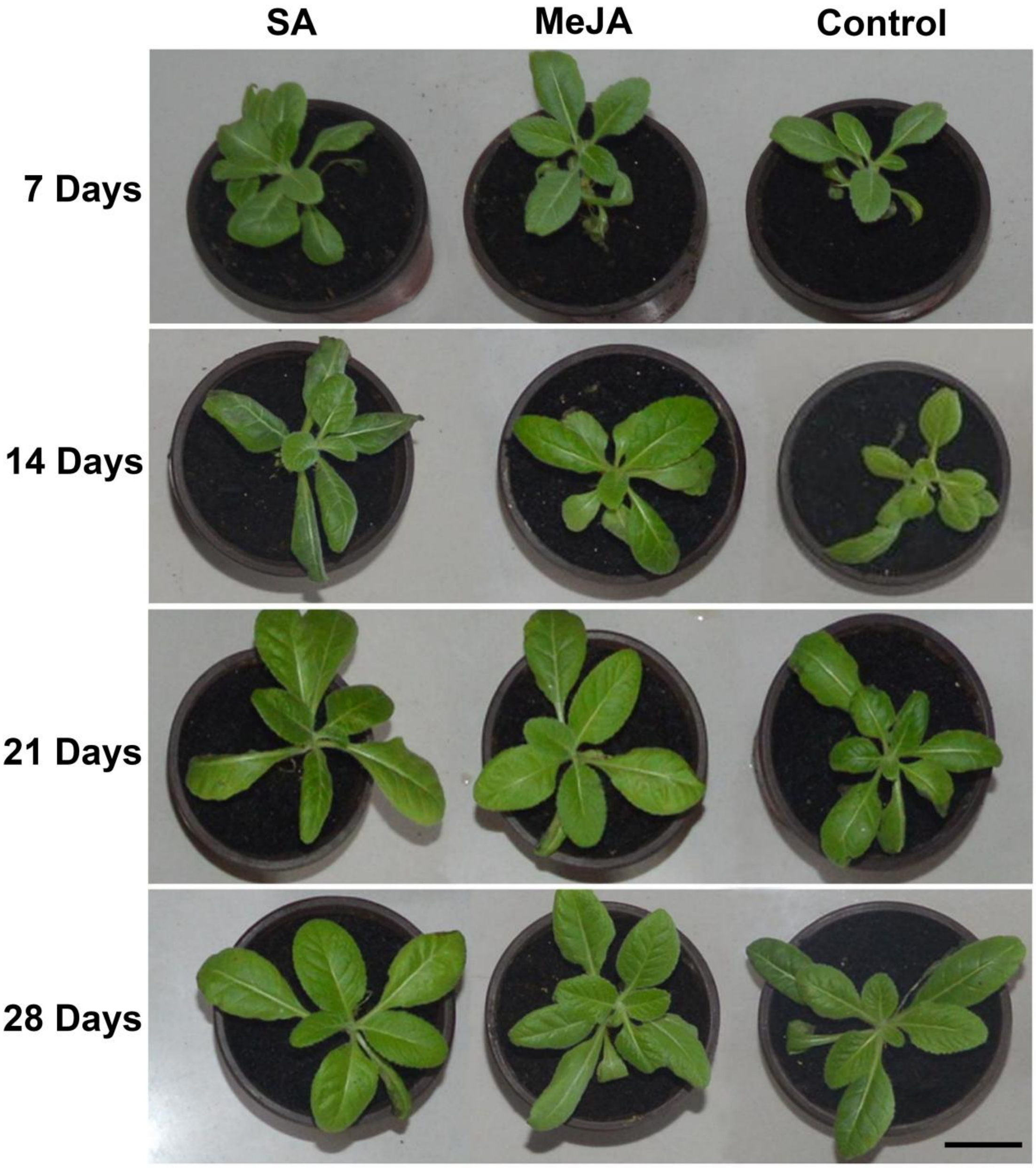

Figure S6 The constructs for various *RgMATE6* vectors. These include the CaMV35S: RgMATE6-GFP (A), RgMATE6-OE (B) and RgMATE6-RNAi (C) constructs.

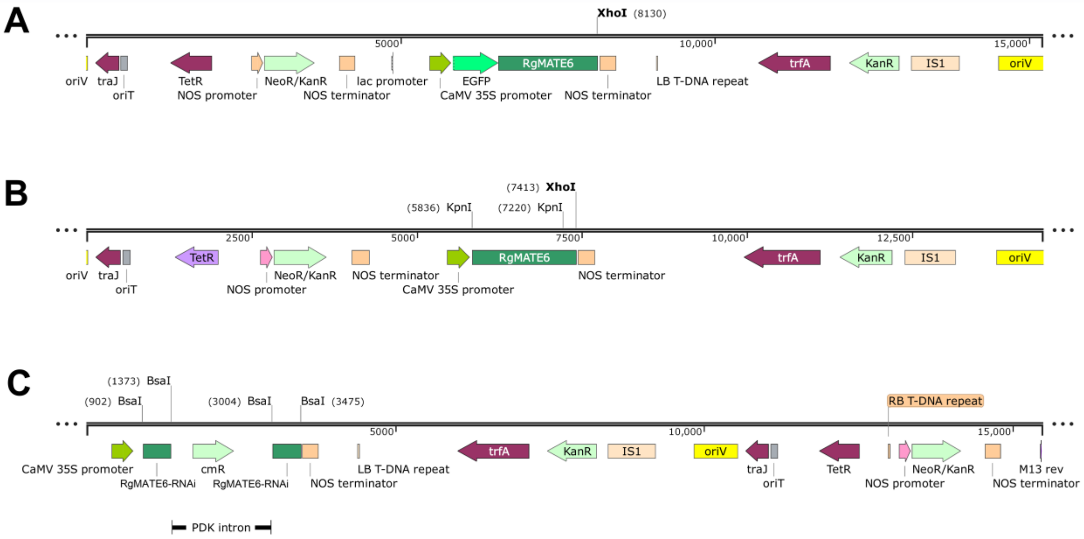

Figure S7 Generation of transgenic *RgMATE6 R. glutinosa* plants. (A) Co-cultivation of the explants and *Agrobacterium tumefaciens*; (B) and (C) the callus induction/selection medium from the explants-transferred; (D) the adventitious shoots induction/selection medium from the callus; (E) and (F) the transgenic seedlings were generated in the selection medium; (G) and (H) acclimatization to organic and field soils (scale bars = 1 cm).

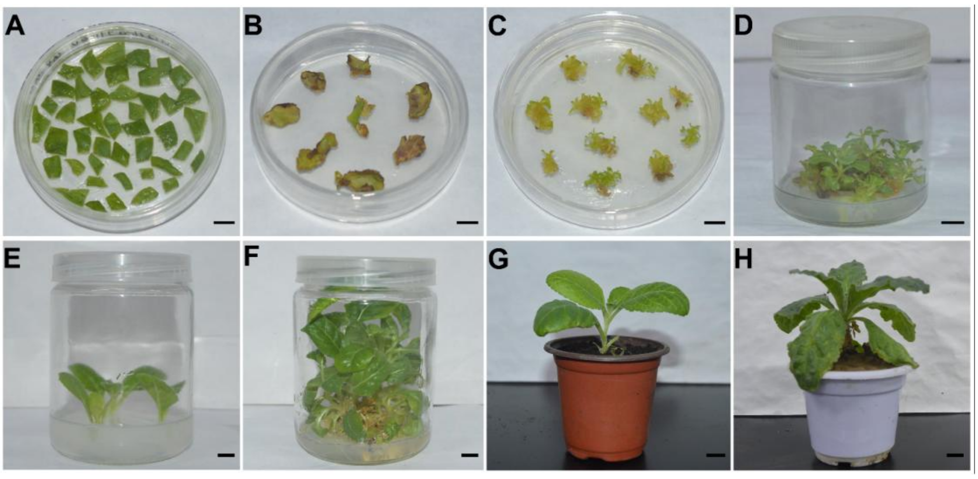

Table S1 The correlation between the *RgMATE6* expression levels and the ACT content of *R. glutinosa* under various treatments was quantified by the Pearson correlation coefficient (*r*). Note: "*" represent significantly different levels of 0.05, respectively.

**Expression levels** / **ACT content from various treatment**	**RgMATE6**
SA treatment	0.954*
MeJA treatment	0.893
Non-treatment (control)	0.86

Table S2 Primer sequences used for qRT-PCR analysis.

Gene name	Genebank ID	Product length (bp)	Forward (5' to 3')	Tm (°C)	Reverse (5' to 3')	Tm (°C)
RgMATE6	MK120918.1	130	CTGCCATAAGTGTAAGAG	56.3	AAGATGAGGAGAAGGATT	56.9
RgMATE12	MK120924.1	103	GATTGAAGGAACAAGTGGAT	59.5	ACCGCTATAACTCTCGTAA	59.1
RgTyDC	OK043668	97	GGGAGAAATGTTGAGCACTGG	58.2	GAGCCAATCCATGACGATGC	57.8
RgCuAO	OR947317	80	TAATAAGACCGAGCAATG	55.4	CCTGTTAGACCATACTTC	55.2
RgPAR	OR947319	98	TTCGGCTAATGGAAGATAC	58.2	CAGGAAGTTGAAGAGTAGG	58.4
RgUGT	OR947322	124	GAGTTGATGATTGGAGAA	55.2	GCACTTGATTGATAACATTA	55.3
RgPPO	OP972605.1	85	GAGTTGCTTGTGCTGGAA	62.1	CATTATCATCCTCGTCGTTCA	61.7
RgHCT	OP556356.1	89	GATTATACTACTCCGATTGATGA	59.3	ATACCGAGACCAATGCTA	58.8
RgActin	EU526396.1	79	GCCATGTATGTTGCTATT	56.5	CACCAGAATCCATCACTA	57.1

Table S3 The primer sequences of *RgMATE6* used to constructs, and the primer sequences of positive screened gene in *R. glutinosa*. Note: the lowercases represent the sequences of these vector adapters.

**Primer type**	**Primer**	**Sequence (5'to 3')**	**T_m (°C)**	**Product length (bp)**
RgMATE6-GFP for expression in *Nicotiana benthamiana*	Forward	ggcatggacgagctgtacaagATGGAGGACAACTCCAAGCAGC	72.6	1575
	Reverse	cggggaaattcgagctctcactcgagTATTTAATTTCTCCAAGTCCTTTGC	70.4	
RgMATE6-OE for overexpression	Forward	ctagaggatccccgggggtaccATGGAGGACAACTCCAAGCAGC	75.4	1575
	Reverse	cggggaaattcgagctctcactcgagTATTTAATTTCTCCAAGTCCTTTGC	68.3	
RgMATE6-RNAi for RNAi repression	Forward	accaggtctcaggagGGTAAAGCGTGGCCTGGATTTAG	60.1	453
	Reverse	accaggtctcatcgtGCTGTTGACCACAATAGAGAAGGC	59.8	
NPTII gene from positive transgenic plants	Forward	GATCTCCTGTCATCTCACCTTGCTCC	61.9	385
	Reverse	CAATATCACGGGTAGCCAACGCTATG	62.1	
